# College of Professional Teachers

**Published:** 1842-07

**Authors:** 


### THE COLLEGE OF PROFESSIONAL TEACHERS.

We have just received the Minutes of the eleventh session of the *Western Literary Institute and College of Professional Teachers,* held in Cincinnati, the place of all the other meetings, in the month of October last. By these proceedings we learn, that the next meeting will be held in the city of Louisville, on Monday, the 15th day of August. As this will be the first meeting in our city, we hope to see it well attended by gentlemen from a distance, not less than our own citizens. Medical gentlemen, who do not reside very distant from us, and can leave their patients, will be well rewarded for an attendance on the meeting. They are deeply interested in the promotion of sound elementary learning, without which we shall not have students qualified for the study of medicine, nor graduates fitted to do honor to the profession. D.

